# Supplementary material for: Multilayer brain networks can identify the epileptogenic zone and seizure dynamics
Source: eLife. 2023 Mar 17;12:e68531. doi: 10.7554/eLife.68531 (PMC10065796; doi:10.7554/eLife.68531)
Supplement: Figure 2—source data 1. — Common channels among the two methods are highlighted. [file elife-68531-fig2-data1.docx]

**Figure 2-source data 1**

| ID | Predicted EZ by mlEVC | Predicted EZ by Fingerprint |
| --- | --- | --- |
| 1 | X01-X02, X02-X03, X03-X04, X05-X06 | R01-R02, R02-R03, R03-R04, **X01-X02, X02-X03, X03-X04, X05-X06** |
| 2 | Full seizure data was not available | |
| 3 | C01-C02, C02-C03 | B01-B02, B02-B03, B03-B04, B04-B05, B07-B08, B08-B09, **C02-C03**, E01-E02, E02-E03, E06-E07 |
| 4 | Lp01-Lp02, Lp02-Lp03, Lp03-Lp04, Lp04-Lp05 | **Lp01-Lp02, Lp03-Lp04**, Lp05-Lp06 |
| 5 | Lp01-Lp02, Lp02-Lp03 | **Lp01-Lp02** |
| 6 | Op06-Op07, Op07-Op08 |  |
| 7 | K01-K02, N01-N02, N02-N03 | M03-M04, M05-M06, M07-M08 |
| 8 |  | O07-O08 |
| 9 | A01-A02, B01-B02, B02-B03, B03-B04, B04-B05, B05-B06 | A03-A04 |
| 10 | O09-O10, O10-O11 | V07-V08 |
| 11 |  | L05-L06, L06-L07, L07-L08 |
| 12 | K05-K06, K06-K07, K07-K08 | K04-K05, **K05-K06, K07-K08** |
| 13 | Xp03-Xp04, Xp04-Xp05, Xp05-Xp06, Xp06-Xp07 | Wp06-Wp07 |
| 14 | R04-R05, R05-R06 | **R04-R05, R05-R06** |
| 15 | L01-L02, L02-L03 | **L01-L02, L02-L03**, L03-L04, L04-L05, L05-L06, O03-O04, O05-O06, Q05-Q06 |
| 16 | A06-A07, A08-A09 |  |
| 17 | Tp01-Tp02, Tp02-Tp03, Tp03-Tp04, Tp04-Tp05, Tp05-Tp06, Tp06-Tp07, Tp07-Tp08, Tp08-Tp09 | Bp01-Bp02, Bp03-Bp04, **Tp01-Tp02, Tp04-Tp05, Tp05-Tp06, Tp07-Tp08, Tp08-Tp09** |
