# Supplementary material for: Multilayer brain networks can identify the epileptogenic zone and seizure dynamics
Source: eLife. 2023 Mar 17;12:e68531. doi: 10.7554/eLife.68531 (PMC10065796; doi:10.7554/eLife.68531)
Supplement: Figure 2—source data 2. — Important note: 16 out of 17 misidentified electrodes in our method are from three patients only. [file elife-68531-fig2-data2.docx]

**Figure 2-source data 2**

|  | **mlEVC** | **Fingerprint** |
| --- | --- | --- |
| **True Positives** | 46 | 51 |
| **False Positives** | 17 | 6 |
