# Supplementary material for: Multilayer brain networks can identify the epileptogenic zone and seizure dynamics
Source: eLife. 2023 Mar 17;12:e68531. doi: 10.7554/eLife.68531 (PMC10065796; doi:10.7554/eLife.68531)
Supplement: Figure 2—source data 3. [file elife-68531-fig2-data3.docx]

**Figure 2-source data 3**

| **Coupling** | **0** | **1** | **2** | **3** | **4** | **5** | **6** | **7** | **8** | **9** | **10** | **Proposed** |
| --- | --- | --- | --- | --- | --- | --- | --- | --- | --- | --- | --- | --- |
| **True Positive**  **(TP)** | 30 | 23 | 42 | 36 | 45 | 57 | 49 | 47 | 36 | 37 | 55 | 46 |
| **False Positive**  **(FP)** | 23 | 34 | 28 | 28 | 31 | 26 | 32 | 33 | 34 | 41 | 33 | 17 |
| **Number of patients with zero TP** | 5 | 8 | 6 | 6 | 5 | 3 | 4 | 5 | 5 | 5 | 4 | 2 |
