# Supplementary material for: Multilayer brain networks can identify the epileptogenic zone and seizure dynamics
Source: eLife. 2023 Mar 17;12:e68531. doi: 10.7554/eLife.68531 (PMC10065796; doi:10.7554/eLife.68531)
Supplement: Figure 4—source data 1. — Post-hoc tests on network measures in high-frequency. p-values smaller than 0.05 are bolded. [file elife-68531-fig4-data1.docx]

**Figure 4-source data 1**

| **Post-hoc analysis** | **Corrected p-values** |
| --- | --- |
| Pre-ictal: EZ vs RnEZ | ≈ 1 |
| Pre-ictal: EZ vs nR | ≈ 1 |
| Pre-ictal: RnEZ vs nR | ≈ 1 |
| Mid-seizure: EZ vs RnEZ | **0.0108** |
| Mid-seizure: EZ vs nR | **< 10^-4^** |
| Mid-seizure: RnEZ vs nR | **< 10^-4^** |
| Post-ictal: EZ vs RnEZ | 0.2664 |
| Post-ictal: EZ vs nR | 0.1680 |
| Post-ictal: RnEZ vs nR | ≈ 1 |
| EZ: Pre-ictal vs Mid-seizure | **0.0020** |
| EZ: Pre-ictal vs Post-ictal | **< 10^-4^** |
| EZ: Mid-seizure vs Post-ictal | **< 10^-4^** |
| RnEZ: Pre-ictal vs Mid-seizure | 0.0560 |
| RnEZ: Pre-ictal vs Post-ictal | **< 10^-4^** |
| RnEZ: Mid-seizure vs Post | **< 10^-4^** |
| nR: Pre-ictal vs Mid-seizure | ≈ 1 |
| nR: Pre-ictal vs Post-ictal | **< 10^-4^** |
| nR: Mid-seizure vs Post-ictal | **< 10^-4^** |
| EZ: predicted epileptogenic zone, RnEZ: resected non-EZ, nR: non-resected | |
